# ve-SEQ: Robust, unbiased enrichment for streamlined detection and whole-genome sequencing of HCV and other highly diverse pathogens

**DOI:** 10.12688/f1000research.7111.1

**Published:** 2015-10-13

**Authors:** David Bonsall, M. Azim Ansari, Camilla Ip, Amy Trebes, Anthony Brown, Paul Klenerman, David Buck, STOP-HCV Consortium, Paolo Piazza, Eleanor Barnes, Rory Bowden

**Affiliations:** 1 Peter Medawar Building for Pathogen Research, Nuffield Department of Medicine, University of Oxford, Oxford, OX1 4BH, UK; 2 Oxford Martin School, University of Oxford, Oxford, OX1 4BH, UK; 3 Oxford Genomics Centre, Wellcome Trust Centre for Human Genetics, University of Oxford, Oxford, OX1 4BH, UK; 4 National Institute for Health Research Biomedical Research Centre, John Radcliffe Hospital, Oxford, UK

**Keywords:** Virus genome sequencing, Sequence capture and enrichment, Anti-viral resistance, Hepatitis C virus

## Abstract

The routine availability of high-depth virus sequence data would allow the sensitive detection of resistance-associated variants that can jeopardize HIV or hepatitis C virus (HCV) treatment. We introduce ve-SEQ, a high-throughput method for sequence-specific enrichment and characterization of whole-virus genomes at up to 20% divergence from a reference sequence and 1,000-fold greater sensitivity than direct sequencing. The extreme genetic diversity of HCV led us to implement an algorithm for the efficient design of panels of oligonucleotide probes to capture any sequence among a defined set of targets without detectable bias. ve-SEQ enables efficient detection and sequencing of any HCV genome, including mixtures and intra-host variants, in a single experiment, with greater tolerance of sequence diversity than standard amplification methods and greater sensitivity than metagenomic sequencing, features that are directly applicable to other pathogens or arbitrary groups of target organisms, allowing the combination of sensitive detection with sequencing in many settings.

## Introduction and background

With a world-wide prevalence estimated at 2.8% ^1, 2^ hepatitis C virus (HCV) poses a global health challenge unrivalled by any curable viral infection. In recent years, direct-acting antiviral (DAA) combination therapies have substantially improved outcomes, but fundamental barriers to eradication remain, including reduced efficacy against genotype 3 infections ^3, 4^ and a cost of modern treatments that is out of reach of even middle-income countries. Newer DAAs such as those targeting HCV’s polymerase and NS5a proteins augment protease inhibitors ^5^, but genotype-limited efficacy and the possibility of resistance mean that HCV genotyping and periodic monitoring of viral load (VL) will remain important in the selection and monitoring of DAA therapies.

Resistance testing by PCR and sequencing of relevant genes is routinely used before initiation of HIV treatment and after its virological failure ^6^. Similar testing in HCV is an exciting prospect, with potential benefits in efficacy and cost. With some notable exceptions, resistance-associated variant (RAV) status at baseline has not been shown to be strongly predictive of treatment success ( https://www.nice.org.uk/guidance/ta331), however the role of resistance testing in informing choice and timing of therapy after HCV treatment failure is an active area of clinical research (e.g. HCV-TARGET ^7^). It is clear from clinical trials in which RAVs were assessed via amplicon sequencing that the relevance of particular mutations depends on both the drug in question and the genetic background of the virus, and attempts have been made to summarise these data as more drugs enter clinical practice ^8^.

As more data is acquired through phase 4, post-marketing studies, our ability to predict treatment success from viral genetic information is likely to improve, leading to higher cure rates across a greater variety of antiviral agents, with potential long-term benefits in treatment cost. However, several questions remain unanswered, including the relevance of variants detected at low frequency within the viral quasispecies and the impact of combinations of mutations on viral fitness, drug susceptibility and the genetic barrier to resistance. To date, these questions have escaped formal investigation owing to the technological challenges in obtaining whole-genome HCV sequences. A complete evaluation of prospective RAV characterization in guiding therapeutic options requires a comprehensive method for high-sensitivity variant detection, for which the development of efficient, unbiased, and cost-effective whole-genome sequencing methods seems a key requirement. Recent advances in genotype-agnostic whole-genome sequencing of HCV have been promising ^9^, but there is still room for improvement in sensitivity, throughput and cost.

HCV strains fall into seven recognized genotypes which differ from each other at an average of 30–35% of nucleotide sites across the ~9650 nt genome ^10^, which is divided into highly conserved and extremely diverse regions of sequence. Genotypes are classified into approximately 67 subtypes, which differ at up to approximately 15% of nucleotide sites and include the globally distributed subtypes 1a, 1b, 2a, and 3a ^10^. Available methods for the characterization of genetically diverse viruses such as HCV in clinical samples present several technical challenges. Amplification of reverse-transcribed virus RNA by PCR relies on a close match between primers and relatively conserved regions of the target, including an absolute match at the 3’ end of each primer, necessitating the design of multiple, genotype-specific sets of overlapping amplicons to recover complete genome sequences. In practical terms, PCR-based whole genome sequencing for HCV is complex and prone to technical failure, requiring a genotyping stage for primer selection, followed by genotype-specific amplification of several fragments and sequencing ^11, 12^, typically using a next-generation platform such as Illumina. The results can include high-depth coverage of the identified genotype, useful for the identification of known drug-related and immune escape variants, but the technique is less appropriate for the detection of low-frequency co-infections, uncovering novel diversity, or high-throughput analysis.

An alternative approach, and the starting point of this research, is a method termed virus RNA-seq ^13^, which efficiently obtains direct “metagenomic” sequence data in the form of Illumina sequence reads from clinical material such as plasma ^14^ and which we used recently to identify a genotype 4 – genotype 1 chimeric isolate from a patient in Cameroon ^15^. Virus RNA-seq is demonstrably unbiased with respect to the detection of any virus genotype, but relatively insensitive and costly for the recovery of whole virus genomes, even with modern sequencing technologies, because in many cases >99% of all sequence data generated derives from the host and is discarded ^9, 13^.

Strategies to deplete host-derived nucleic acids in virus metagenomic whole-genome sequencing have been applied successfully but are intrinsically limited in their effectiveness by the often-variable characteristics of the input sample. Using DNAase digestion of plasma before reverse transcription-based RNA amplification and a modified low-input Illumina library preparation, HCV-specific read proportions of 1.5%–47.7% have been reported ^9^, for samples with relatively high VLs (>1.8 10 ^5^ IU/ml), sequenced in small multiplexes of eight samples per Illumina MiSeq run. Oligonucleotide-targeted RNAse H digestion of host rRNA has been used to improve the yield of Lassa and Ebola virus sequences but virus-specific sequencing efficiency remains close to 1% ^16^. More promisingly, enrichment using biotinylated probes that target viral sequences has significantly improved sensitivity and efficiency of herpesvirus ^17^, Lassa virus ^16^ and *Mycobacteria tuberculosis* ^18^ sequencing.

The ideal methodology for one-step, high-throughput clinical virus sequencing would combine the benefits of high-throughput sequencing with the sensitivity of PCR, while avoiding the pitfalls of PCR-based amplification and the inefficiencies of RNA-seq based metagenomic approaches. We report a comprehensive approach to virus-specific, genotype-agnostic, probe-based enrichment and sequencing of whole HCV genomes at a depth sufficient to call minor variants without bias and at a cost compatible with routine clinical HCV genotyping, that in principle can also be applied to other pathogens.

## Materials and methods

### Sample collection and preparation

Samples for optimization of sequencing methods were acquired from HCV Research UK ( http://www.hcvresearchuk.org/), whose clinical samples were used with informed consent, conforming to the ethical guidelines of the 1975 Declaration of Helsinki. Study protocols were approved by the NRES Committee East Midlands, Derby (Ethics reference 11/EM/0323). Samples for resistance testing were obtained from patients enrolled and consented as part of the OxBRC Prospective Cohort Study in Hepatitis C (Ethics reference 09/H0604/20) at the Oxford University Hospitals NHS Trust.

Patient plasma was collected from EDTA blood tubes by centrifugation for 10 minutes at 600g in a Heraeus Megafuge, and stored at -80°C. RNA was isolated from 500µl plasma volumes using the NucliSENS magnetic extraction system (bioMerieux) and collected in 30µl of kit elution buffer for storage in aliquots at -80°C.

### Sequencing library construction, enrichment and sequencing

Libraries were prepared for Illumina sequencing using the NEBNext ^®^ Ultra™ Directional RNA Library Prep Kit for Illumina ^®^ (New England Biolabs) with 5µl sample (maximum 10ng total RNA) and previously published modifications of the manufacturer’s guidelines (v2.0) ^13^, briefly: fragmentation for 5 or 12 minutes at 94°C, omission of Actinomycin D at first-strand reverse transcription, library amplification for 15–18 PCR cycles using custom indexed primers ^19^ and post-PCR clean-up with 0.85× volume Ampure XP (Beckman Coulter).

Libraries were quantified using Quant-iT™ PicoGreen ^®^ dsDNA Assay Kit (Invitrogen) and analysed using Agilent TapeStation with D1K High Sensitivity kit (Agilent) for equimolar pooling, then re-normalized by qPCR using the KAPA SYBR ^®^ FAST qPCR Kit (Kapa Biosystems) for sequencing. Metagenomic virus RNA-Seq libraries were sequenced with 100b paired-end reads on the Illumina HiSeq 2500 with v3 Rapid chemistry.

A 500ng aliquot of the pooled library was enriched using the xGen ^®^ Lockdown ^®^ protocol from IDT (Rapid Protocol for DNA Probe Hybridization and Target Capture Using an Illumina TruSeq ^®^ or Ion Torrent ^®^ Library (v1.0), Integrated DNA Technologies) with equimolar-pooled 120nt DNA oligonucleotide probes (IDT) followed by a 12-cycle, modified, on-bead, post-enrichment PCR re-amplification. The cleaned post-enrichment ve-Seq library was normalized with the aid of qPCR and sequenced with 100b paired-end reads on a single run of the Illumina MiSeq using v2 chemistry.

### Sequence data analysis

De-multiplexed sequence read-pairs were trimmed of low-quality bases using QUASR v7.01 ^20^ and adapter sequences with CutAdapt version 1.7.1 ^21^ and subsequently discarded if either read had less than 50b remaining sequence or if both reads matched the human reference sequence using Bowtie version 2.2.4 ^22^. The remaining read pool was screened against a BLASTn database containing all 165 ICTV (International Committee on the Taxonomy of Viruses) HCV genomes ( http://talk.ictvonline.org/ictv_wikis/m/files_flavi/default.aspx) both to choose an appropriate reference and to select those reads which formed a majority population for *de novo* assembly with Vicuna v1.3 ^23^ and finishing with V-FAT v1.0 ( http://www.broadinstitute.org/scientific-community/science/projects/viral-genomics/v-fat). Reads were mapped back to the assembly using Mosaik v2.2.28 ^24^, variants were called by V-Phaser v2.0 ^25^ and intra-host diversity was explored with V-Profiler v1.0 ^26^.

## Results

### Virus RNA-seq detection of RNA viruses in plasma

**Figure 1.**
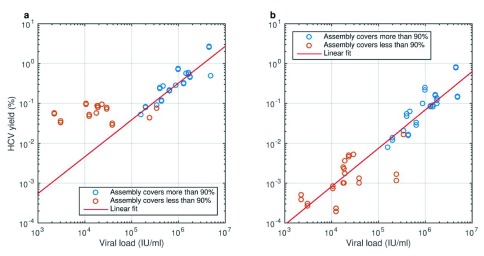
HCV metagenomic sequence yield is proportional to viral load. The yield of reads that map to any HCV genome and the probability of successful *de novo* assembly of a complete genome sequence both depend on viral load (VL). Samples were prepared as replicate libraries that were sequenced simultaneously with consistent yield. Blue circles: successful *de novo* assembly (>90% complete genome length recovered); red circles: incomplete genome assembly. **a**. With standard mapping criteria, up to 2.8% of reads match HCV and a background 0.02–0.1% of low-complexity human-derived sequences overwhelms the HCV signal in low-VL samples. Linear trend is plotted for samples with VL > 10 ^5^ IU/ml. **b**. Under stringent mapping criteria (mapping Q > 40), lower complexity human and HCV reads are excluded and yield is proportional to VL (slope of linear trend in log-log space not significantly different from 1) across the VL range.

We first evaluated the performance of a conventional, “metagenomic” approach to virus whole-genome sequencing ^13^. Indexed sequencing libraries were constructed in duplicate from plasma RNA of 29 subjects infected with diverse HCV subtypes (1a, 1b, 2a, 2b, 3a, 4a and 4d) and a 3.5-log range of VLs (2,200–4.9 million IU/mL; 1 IU = 2.7 copies on the instrument we use) and sequenced on a single Illumina HiSeq 2500 Rapid run, producing a median of 8.0 million reads per sample (range 6.0–24.9 million), of which 0.37% originated from HCV (range 0.03%–2.8%) ( Supplementary Table S1). There was a linear relationship between HCV VL and the yield of HCV reads with high mapping quality ( Figure 1). Mapping the HCV reads for each sample to the closest available reference (either a database reference or a *de novo* assembly of the same reads) produced patterns of peaks and troughs in sequence coverage along the genome that showed some similarity between samples of different subtypes and were highly reproducible between library and sequencing technical replicates; we therefore infer patterns of coverage are caused mainly by genomic features such as secondary structure and melting temperature ^27^.

In its standard form, metagenomic sequencing of a batch of up to 96 samples costs <£100 per sample. In this experiment, a VL of approximately 2×10 ^5^ IU/mL was sufficient to attain a mean read depth across the genome of ~30 and a high probability of successful *de novo* assembly, but higher read depths are necessary for precise characterization of minor variants. Results are better with high-VL samples, and measures to increase library complexity and improve release of virus during RNA isolation may improve variant-calling sensitivity, but the low efficiency of metagenomic sequencing poses a fundamental problem.

### ve-Seq: Probe-based enrichment increases HCV sequence yield

When the sequence of interest comprises only a small fraction of the starting material, probe-based sequence capture, as used in exome sequencing, can dramatically increase sequencing efficiency ^17, 28^. Anticipating the challenge posed by the extreme diversity of HCV, we drew on a representative genome sequence from each of four common genotypes (1a, 2b, 3a and 4a) to construct a combined panel of biotinylated DNA oligonucleotides (xGen ^®^ Lockdown ^®^ probes, IDT) comprising four sets of 155–157 probes, each a 120 nt sequence fragment overlapping the next by 60 nt, and excluding the 3’ poly-(U) tract to avoid enrichment of low-complexity non-HCV sequences.

We enriched the previously-sequenced pool of libraries for HCV sequences by solution hybridization with the 4-genotype probe panel and sequenced it on the Illumina MiSeq platform. This yielded a greater-than 16 × increase in the total number of HCV reads produced, even with an output of ~14 × fewer reads than the previous metagenomic sequencing on the higher-output HiSeq ( Supplementary Table S1). HCV sequence content reached 86% in the enriched pool (range 1–98% among samples), equivalent to a median 1,660 (range 10–75,700) genomic average read depth or >10 ^3^-fold enrichment for samples with mid-range VL ( Supplementary Figure S1); and hit saturation point (near-100% HCV reads) for samples with higher starting HCV content. Although probe panels can be expensive to synthesize, they can be used for many (hundreds of) pooled captures, so the lower sequencing costs in ve-SEQ more than account for the extra costs of the enrichment step.

### Probe-target dissimilarity reduces enrichment efficiency

**Figure 2.**
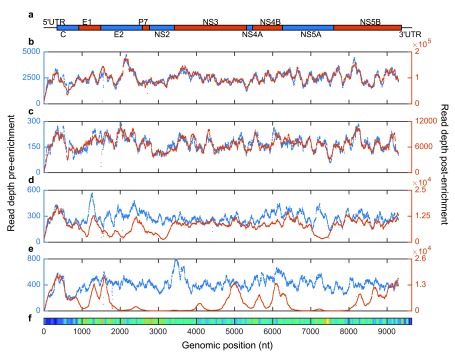
Enrichment efficiency decreases with phylogenetic distance. Read depth across the genome before (blue, left axis) and after (red, right axis) enrichment with a single-sequence subtype 1a probe set. **a**. The HCV genome comprises 5’ and 3’ untranslated regions (UTRs) and a large central segment encoding a single polyprotein that is cleaved into ten proteins. **b**. A subtype 1a sample enriched with probes derived from its own consensus sequence yields coverage patterns across the genome essentially identical to metagenomic sequencing. **c**. A distinct subtype 1a sample produces highly similar but non-identical patterns of pre- and post-enrichment genomic coverage. **d**. A subtype 1b sample yields low read depths at loci that are relatively divergent from the 1a probe sequence (E1, E2, NS2 and NS5a). **e**. Sequence capture of a sample from a different genotype, 3a, is poor across large segments of the genome. **f**. Heat map representing average diversity (calculated as Shannon entropy) among 165 HCV reference genomes. Nucleotide diversity varies dramatically across the genome and tracks drops in enrichment efficiency between phylogenetically distinct probe-target combinations.

We used a single-genome, subtype 1a subset of the 4-genotype probe panel to investigate the effect of varying probe-target sequence identity on ve-SEQ enrichment success ( Figure 2). When a sample is enriched with probes derived from that sample’s consensus sequence, there is no detectable bias in read depth with genomic position (i.e. coverage across the genome for enriched data follows a pattern almost identical to unenriched data, albeit at much higher read depth). When a non-identical sample of the same subtype is enriched, coverage patterns coincide, but are not identical. When a sample from the same genotype but a different subtype to the probe panel is enriched, large sections of the genome are adequately sequenced, but the most divergent regions are covered poorly and whole-genome assembly fails for samples with low viral load ( Supplementary Table S1). When the sample and the enrichment probe set come from different genotypes, only the most conserved parts of the genome are adequately represented with ve-SEQ data and read depth is essentially zero for divergent regions.

**Figure 3.**
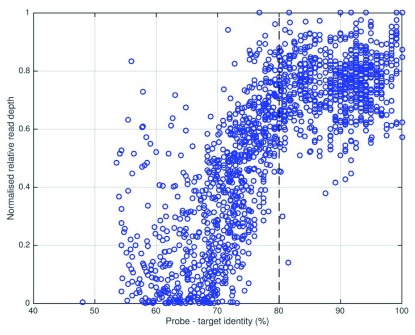
Enrichment efficiency is directly related to probe-target identity. A set of 10 HCV samples with highest VL was sequenced before and after enrichment with a single-genome, subtype 1a probe set, and for each sample the relative read depth for each probe window was plotted against the maximum identity between target and any probe. Read depth ratio was normalized by giving the most efficiently enriched probe position (in the highly conserved 5’ UTR) a value of 1. Maximal enrichment is observed where probe-target identity exceeds approximately 80% and enrichment decreases dramatically as identity falls below 80%.

In order to rationalize our approach to probe choice and enable the design of an efficient, comprehensive HCV enrichment probe set, we analysed the relationship between probe-target similarity and the relative efficiency of enrichment ( Figure 3). Noting a strong inflection point, we deduced that a minimum 80% identity between a 120 nt segment of sample sequence and its closest matching probe was sufficient to ensure near-maximal enrichment, assuming that each sequencing library molecule interacted with a single probe molecule and ignoring the potential effects of bridging capture (i.e. successful sequencing of a poorly matching fragment effected by hybridization of an adjacent target sequence on the same library molecule to a better-matching probe). The 20% divergence cutoff for successful enrichment falls between the mean inter-subtype (<15%) and inter-genotype (30–35%) divergence levels, explaining why enrichment with a subtype-mismatched probe set leads to only localized bias, while genotype-mismatch results in failure across most of the genome. It also follows from this analysis that when enrichment is performing well, there should be no detectable bias in the representation of single nucleotide variant alleles such as RAVs.

### Design of a comprehensive probe set for HCV

As is evident from the previous section, a probe panel based on just four subtype-representative sequences cannot perfectly capture HCV global diversity. Exploiting the observation that some regions of the HCV genome (e.g. the 5’UTR) are well-enough conserved to not require multiple probe sets, together with the 20% divergence cutoff for efficient capture, we implemented an algorithm for efficient probe set design that would facilitate a comprehensive HCV enrichment panel as well as, in principle, efficient probe sets for other organisms.

We started with the 4-genotype probe panel and added extra probes to improve coverage for already-included subtypes 1a, 2b, 3a and 4a as well as the extra subtypes 1b, 2a, 2c, 5a and 6a, using a database of 482 reference whole-genome sequences. First we calculated a consensus sequence for each subtype. Then, starting with the existing probe set and the first genome in the most common subtype (1b), we identified genomic regions with less than 80% identity to any of the probes already in the panel. For each such region the subtype consensus sequence was considered as a potential probe but only used if it was ≥80% identical to the genomic sequence it replaced; otherwise the genomic sequence fragment was added as a new probe. The process was repeated for each 1b reference sequence and then similarly for each subtype.

In contrast to the naïve design of probe sets with the standard IDT approach that requires 155–157 probes per HCV target genome, we were able to augment our 4-genome probe panel to represent the known diversity of nine subtypes spanning six of the seven recognized genotypes with only another 491 probes (1,116 total). Our algorithm substantially and automatically reduces redundancy: a completely naïve approach that simply encoded every genome in the reference set, without accounting for similarity between genomes, would have dictated a prohibitively expensive set of ~75,000 probes. In contrast, if we had instead started from scratch, we estimate that our simple algorithm could have produced an equally effective combined panel for nine subtypes with as few as 955 probes. In informal testing, a typical sample from the newly added subtype 1b achieved near-zero bias even though its exact sequence was not encoded in the probe set but was instead covered by reference to recorded sequence diversity ( Supplementary Figure S2), and a sample from subtype 4d, not included in the revised probe set, achieved adequate although imperfect enrichment ( Supplementary Figure S3), consistent with previous subtype-mismatched captures. Although feasible and relatively inexpensive, we have deferred the addition of probes for remaining rare subtypes.

### Detection of resistance-associated variants in clinical samples

To explore the potential utility of high-depth RAV data in predicting the clinical effectiveness of HCV treatment, we used ve-SEQ to analyse retrospectively plasma samples collected from 33 genotype 1-infected patients before NS3-targeting DAA therapy with Boceprevir (14 patients) or Telaprevir (19 patients) ( Supplementary Table S2). We obtained whole-genome sequences for all samples, with a mean read depth of 4600 across the NS3 gene. We first confirmed that our sequence data (28 subtype 1a and 5 subtype 1b) matched clinical subtyping data where the latter was available.

Mutations in the NS3 gene, denoted T54S and V55I, were detected in patient P23, in whom Boceprevir treatment failed to suppress HCV. Only one other patient had relevant baseline resistance: P6 possessed a single T54S mutation, yet cleared infection with 48 weeks of BCP. Additionally, Simeprevir RAVs Q80K/R were detected in five patients with genotype 1a virus, consistent with the reported prevalence of these mutations in PI-naïve patients ^29^. Variants associated with NS5A inhibitor resistance were detected in 11 patients, including nine with combinations of two or more RAVs, previously associated with higher relapse rates than Lidipesvir/Sofosbuvir ^30^.

**Figure 4.**
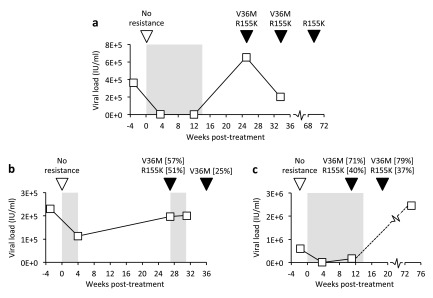
Detection of resistance-associated variants after DAA treatment failure. VL and RAV status for three patients who failed to achieve sustained virological response after Telaprevir-based therapy. Grey shading: duration of therapy (weeks starting at time 0); squares: VL measurements; inverted triangles: samples sequenced using the comprehensive probe panel (open: no Telaprevir RAVs detected, black: RAVs and supporting read proportions, where <100%).

In samples taken after treatment cessation, five patients carried both V36M and R155K NS3 variants, associated with drug resistance but also reduced virus fitness in the absence of treatment ^31, 32^, including three patients illustrated in Figure 4. RAVs V36M and R155K were each detected independently of the other (in P30 and P33, respectively) and virus sampled in P27 during treatment revealed approximately 2-fold more V36M variants than R155K, confirming that V36M alone was sufficient to confer resistance on individual genomes. Telaprevir had failed to suppress virus in subject P24 by week 4 when V36M and R155K variants circulated in approximately half of virus. It is therefore not surprising that a subsequent treatment attempt also failed, providing a real-world clinical example of where sequencing might have prevented futile retreatment. Six weeks after the second treatment attempt had failed, the R155K mutation had reverted to the wild-type arginine residue in all sequence reads. Partial reversion was also observed in P18, although in this instance, reversion of V36M occurred some 20 or more weeks after the cessation of treatment and R155K was still present in 100% of variants 1 year later.

## Discussion

Our ve-SEQ method provides improvements over other approaches currently used for rapid, high-throughput, high-sensitivity characterization of complete virus sequences from clinical samples. These advantages include sequencing efficiency for low-VL samples not available from metagenomic approaches ^9^ and robustness to extreme sequence diversity such as that found in HCV that is not available from PCR-based methods ^8^. Our approach is similar to published methods ^16– 18, 28^ but benefits from low enrichment costs and defined performance that come from efficient probe design and non-proprietary, high throughput sample processing.

In this study, treatment-naïve individuals carried RAVs to NS3 and NS5A inhibitors and emerging resistance was shown to persist 1 year after treatment failure, which stands to complicate empiric selection and timing of HCV treatment, particularly in previously treated patients. Stratification by viral genotype is currently the best strategy for successful treatment; ve-SEQ performs as well as current routine subtyping techniques at comparable cost while additionally offering high-depth, high-throughput and unbiased detection of RAVs, enabling future large-scale evaluation of resistance testing in clinical studies and offering the possibility of replacing current practice with a single highly informative test. Our preliminary analyses reveal cases in which such data may be clinically useful, and the cost of the test compared with that of a failed DAA treatment (e.g. ~£40K for HARVONI ^®^, https://www.nice.org.uk/guidance/gid-tag484) suggests potential for ve-SEQ to be cost-effective in a clinical setting.

Our general approach also has clear application in the detection and sequencing in a single protocol of other pathogens – none is as diverse as HCV – including the potential for multi-pathogen, sub-genomic panels that might replace multiplex PCR-based screening and diagnostic techniques with more comprehensive, higher resolution data at comparable sensitivity ^33^. ve-SEQ works at high-throughput scales, with a standard, plate-based format that makes it affordable and comparable in overall cost to less informative assays. To avoid turnaround delays while maintaining efficiency for routine use, in principle the HCV assay could be combined with assays for other pathogens, and plasma RNA-seq libraries could be pooled with RNA- and DNA-originating libraries from other sample types, for a routine test run on sequencing platforms like the Illumina MiSeq, that are becoming more generally available in large-hospital diagnostic labs. The more a pool of libraries is enriched, the more individual library complexity (broadly, the number of starting molecules of HCV included) becomes important: since the ve-SEQ approach can be used with any library methodology we have now turned our attention to ways of optimizing the yield of HCV in plasma RNA, increasing the amount of library input material and improving library efficiency.

The robustness of probe-based enrichment provides a practical alternative to PCR and similar amplification-based approaches that require a close match between primer and target. We envisage that enrichment could provide almost-hypothesis-free detection for all plausibly present pathogens in clinical samples, both for low-diversity target genomes in which a single representative probe set is sufficient, and by using algorithms such as the one we implement here to efficiently capture more diverse pathogens. Because less sequencing effort is required, the overall cost of an enrichment-based protocol is lower than that of a no-enrichment approach and achieves a greater yield of useful data, more efficiently and robustly than PCR.

## Data availability

The data referenced by this article are under copyright with the following copyright statement: Copyright: © 2015 Bonsall D et al.

Data associated with the article are available under the terms of the Creative Commons Zero "No rights reserved" data waiver (CC0 1.0 Public domain dedication). 

Sequence data, filtered to remove human reads, is available from the European Nucleotide Archive (ENA) under accession PRJEB9338.

## Supplementary materials

**Supplementary Figure S1. Relationship between VL and HCV enrichment ratio.**

Aliquots of the same library pool were sequenced before and after enrichment with a 4-genome (subtypes 1a, 2b, 3a and 4a) enrichment probe set and the ratio of post-enrichment to metagenomic HCV yield (% HCV-matching reads) was plotted against VL. Under stringent mapping criteria (mapping Q > 40) that exclude low-complexity human and HCV sequences, enrichment exceeds 10 ^3^ × for low- to medium-VL samples. For high-VL samples where post-enrichment yield approaches saturation (100%), the achievable enrichment ratio decreases. For such samples, metagenomic sequencing yield is often around 1% in any case.

.

**Supplementary Figure S2. Enrichment of a 1b sample.**

Read depth across the genome before (blue, left axis) and after (red, right axis) enrichment of a subtype 1b sample with each of the three probesets.

**a.** 1a probeset: moderate coverage with genotype- but not subtype-specific probes.

**b.** 1a, 2b, 3a and 4a pooled probeset: similar levels of enrichment to (a) with a probeset that includes single genomes of four genotypes but still does not include 1b.

**c.** 1a, 1b, 2a, 2b, 2c, 3a, 4a, 5a and 6a pooled probeset: almost-unbiased enrichment with a probeset of nine subtypes, including optimized coverage of 1b but not an exact match between probes and the consensus sequence of this sample.

.

**Supplementary Figure S3. Enrichment of a 4d sample.**

Read depth across the genome before (blue, left axis) and after (red, right axis) enrichment of a subtype 4d sample with each of the three probesets.

**a.** 1a probeset: poor coverage with no genotype-specific probes

**b.** 1a, 2b, 3a and 4a pooled probeset: reduced bias in enrichment with genotype- but not subtype-matching probes

**c.** 1a, 1b, 2a, 2b, 2c, 3a, 4a, 5a and 6a pooled probeset: similar levels of enrichment to (b) with a probeset that includes nine subtypes but still does not include 4d.

**Supplementary tables S1 and S2.**

Supplementary tables S1: HCV sequencing statistics and S2: Pre- and post-treatment resistance detected by ve-SEQ

## References

[1] Mohd Hanafiah K Groeger J Flaxman AD: Global epidemiology of hepatitis C virus infection: new estimates of age-specific antibody to HCV seroprevalence. *Hepatology.* 2013;57(4):1333–1342. 10.1002/hep.26141 23172780

[2] Messina JP Humphreys I Flaxman A: Global distribution and prevalence of hepatitis C virus genotypes. *Hepatology.* 2015;61(1):77–87. 10.1002/hep.27259 25069599 PMC4303918

[3] Jacobson IM Gordon SC Kowdley KV: Sofosbuvir for hepatitis C genotype 2 or 3 in patients without treatment options. *N Engl J Med.* 2013;368(20):1867–1877. 10.1056/NEJMoa1214854 23607593

[4] Lawitz E Mangia A Wyles D: Sofosbuvir for previously untreated chronic hepatitis C infection. *N Engl J Med.* 2013;368(20):1878–1887. 10.1056/NEJMoa1214853 23607594

[5] Shah N Pierce T Kowdley KV: Review of direct-acting antiviral agents for the treatment of chronic hepatitis C. *Expert Opin Investig Drugs.* 2013;22(9):1107–1121. 10.1517/13543784.2013.806482 23735127

[6] Asboe D Aitken C Boffito M: British HIV Association guidelines for the routine investigation and monitoring of adult HIV-1-infected individuals 2011. *HIV Med.* 2012;13(1):1–44. 10.1111/j.1468-1293.2011.00971.x 22171742

[7] Gordon SC Muir AJ Lim JK: Safety profile of boceprevir and telaprevir in chronic hepatitis C: real world experience from HCV-TARGET. *J Hepatol.* 2015;62(2):286–293. 10.1016/j.jhep.2014.08.052 25218788 PMC4586075

[8] Hutchison C Kwong A Ray S: Accelerating drug development through collaboration: the Hepatitis C Drug Development Advisory Group. *Clin Pharmacol Ther.* 2014;96(2):162–165. 10.1038/clpt.2014.113 24853733

[9] Hedskog C Chodavarapu K Ku KS: Genotype- and Subtype-Independent Full-Genome Sequencing Assay for Hepatitis C Virus. *J Clin Microbiol.* 2015;53(7):2049–59. 10.1128/JCM.02624-14 25878342 PMC4473213

[10] Smith DB Bukh J Kuiken C: Expanded classification of hepatitis C virus into 7 genotypes and 67 subtypes: updated criteria and genotype assignment web resource. *Hepatology.* 2014;59(1):318–327. 10.1002/hep.26744 24115039 PMC4063340

[11] Humphreys I Fleming V Fabris P: Full-length characterization of hepatitis C virus subtype 3a reveals novel hypervariable regions under positive selection during acute infection. *J Virol.* 2009;83(22):11456–11466. 10.1128/JVI.00884-09 19740991 PMC2772701

[12] Lauck M Alvarado-Mora MV Becker EA: Analysis of hepatitis C virus intrahost diversity across the coding region by ultradeep pyrosequencing. *J Virol.* 2012;86(7):3952–3960. 10.1128/JVI.06627-11 22278255 PMC3302523

[13] Batty EM Wong TH Trebes A: A modified RNA-Seq approach for whole genome sequencing of RNA viruses from faecal and blood samples. *PLoS One.* 2013;8(6):e66129. 10.1371/journal.pone.0066129 23762474 PMC3677912

[14] Ninomiya M Ueno Y Funayama R: Use of Illumina deep sequencing technology to differentiate hepatitis C virus variants. *J Clin Microbiol.* 2012;50(3):857–866. 10.1128/JCM.05715-11 22205816 PMC3295113

[15] Iles JC Njouom R Foupouapouognigni Y: Characterization of Hepatitis C Virus Recombination in Cameroon by Use of Nonspecific Next-Generation Sequencing. *J Clin Microbiol.* 2015;53(10):3155–64. 10.1128/JCM.00483-15 26202126 PMC4572555

[16] Matranga CB Andersen KG Winnicki S: Enhanced methods for unbiased deep sequencing of Lassa and Ebola RNA viruses from clinical and biological samples. *Genome Biol.* 2014;15(11):519. 10.1186/PREACCEPT-1698056557139770 25403361 PMC4262991

[17] Depledge DP Palser AL Watson SJ: Specific capture and whole-genome sequencing of viruses from clinical samples. *PLoS One.* 2011;6(11):e27805. 10.1371/journal.pone.0027805 22125625 PMC3220689

[18] Brown AC Bryant JM Einer-Jensen K: Rapid Whole-Genome Sequencing of *Mycobacterium tuberculosis* Isolates Directly from Clinical Samples. *J Clin Microbiol.* 2015;53(7):2230–7. 10.1128/JCM.00486-15 25972414 PMC4473240

[19] Lamble S Batty E Attar M: Improved workflows for high throughput library preparation using the transposome-based Nextera system. *BMC Biotechnol.* 2013;13:104. 10.1186/1472-6750-13-104 24256843 PMC4222894

[20] Gaidatzis D Lerch A Hahne F: QuasR: quantification and annotation of short reads in R. *Bioinformatics.* 2015;31(7):1130–2. 10.1093/bioinformatics/btu781 25417205 PMC4382904

[21] Martin M: Cutadapt removes adapter sequences from high-throughput sequencing reads. *EMBnet journal.* 2011;17(1): Next Generation Sequencing Data Analysis. 10.14806/ej.17.1.200

[22] Langmead B Salzberg SL: Fast gapped-read alignment with Bowtie 2. *Nat Methods.* 2012;9(4):357–359. 10.1038/nmeth.1923 22388286 PMC3322381

[23] Yang X Charlebois P Gnerre S: *De novo* assembly of highly diverse viral populations. *BMC Genomics.* 2012;13:475. 10.1186/1471-2164-13-475 22974120 PMC3469330

[24] Lee WP Stromberg MP Ward A: MOSAIK: a hash-based algorithm for accurate next-generation sequencing short-read mapping. *PLoS One.* 2014;9(3):e90581. 10.1371/journal.pone.0090581 24599324 PMC3944147

[25] Yang X Charlebois P Macalalad A: V-Phaser 2: variant inference for viral populations. *BMC Genomics.* 2013;14:674. 10.1186/1471-2164-14-674 24088188 PMC3907024

[26] Henn MR Boutwell CL Charlebois P: Whole genome deep sequencing of HIV-1 reveals the impact of early minor variants upon immune recognition during acute infection. *PLoS Pathog.* 2012;8(3):e1002529. 10.1371/journal.ppat.1002529 22412369 PMC3297584

[27] Dohm JC Lottaz C Borodina T: Substantial biases in ultra-short read data sets from high-throughput DNA sequencing. *Nucleic Acids Res.* 2008;36(16):e105. 10.1093/nar/gkn425 18660515 PMC2532726

[28] Melnikov A Galinsky K Rogov P: Hybrid selection for sequencing pathogen genomes from clinical samples. *Genome Biol.* 2011;12(8):R73. 10.1186/gb-2011-12-8-r73 21835008 PMC3245613

[29] Paolucci S Fiorina L Piralla A: Naturally occurring mutations to HCV protease inhibitors in treatment-naïve patients. *Virol J.* 2012;9:245. 10.1186/1743-422X-9-245 23095680 PMC3493344

[30] Afdhal N Reddy KR Nelson DR: Ledipasvir and sofosbuvir for previously treated HCV genotype 1 infection. *N Engl J Med.* 2014;370(16):1483–1493. 10.1056/NEJMoa1316366 24725238

[31] Susser S Welsch C Wang Y: Characterization of resistance to the protease inhibitor boceprevir in hepatitis C virus-infected patients. *Hepatology.* 2009;50(6):1709–1718. 10.1002/hep.23192 19787809

[32] Zhou Y Bartels DJ Hanzelka BL: Phenotypic characterization of resistant Val ^36^ variants of hepatitis C virus NS3-4A serine protease. *Antimicrob Agents Chemother.* 2008;52(1):110–120. 10.1128/AAC.00863-07 17938182 PMC2223918

[33] Cheval J Sauvage V Frangeul L: Evaluation of high-throughput sequencing for identifying known and unknown viruses in biological samples. *J Clin Microbiol.* 2011;49(9):3268–3275. 10.1128/JCM.00850-11 21715589 PMC3165575

