# Supplementary material for: ve-SEQ: Robust, unbiased enrichment for streamlined detection and whole-genome sequencing of HCV and other highly diverse pathogens
Source: F1000Res. 2015 Oct 13;4:1062. [Version 1] doi: 10.12688/f1000research.7111.1 (PMC4821293; doi:10.12688/f1000research.7111.1)
Supplement: Supplementary file 4 [file f1000research-4-7657-s0003.tgz › 3be625c8-17ff-4326-81d3-943f7cd8671f.docx]

**Supplementary Table 1: HCV sequencing statistics**

**Supplementary Table 2: Pre and post treatment resistance detected by ve-SEQ**

| Pt No. | Genotype | | | Drug | Outcome | Pre-treatment VL | Treatment duration (wks) | Reason for Stopping early | Average NS3 read depth | Resistance associated variants (RAVs) pre-treatment | | | Novel RAVs Post-treatment  (NS3) |
| --- | --- | --- | --- | --- | --- | --- | --- | --- | --- | --- | --- | --- | --- |
|  | Clinic | | (WGS) |  |  |  |  |  |  | NS3  (Bold: to prescribed PI) | NS5a | NS5B |  |
| P1 | 1 | (1a) | | TPV | SR | 1.1E6 | 24 |  | 3038 |  |  |  | - |
| P2 | 1 | (1a) | | BCP | SR | 8.7E5 | 48 |  | 452 | Q41H*, Q80R (21%) |  |  | - |
| P3 | 1a | (1a) | | BCP | SR | 1.3E6 | 48 |  | 1223 |  | K24R |  | - |
| P4 | 1a | (1a) | | BCP | SR | 3.7E6 | 48 |  | 1314 |  |  | L112T (67%), M426V | - |
| P5 | 1a | (1a) | | TPV | SR | 2.4E6 | 24 |  | 7086 | Q80K (45%) |  | M426L | - |
| P6 | 1b | (1b) | | BCP | SR | 1.5E6 | 28 |  | 833 | **T54S (87%)** |  | C316N, M426A | - |
| P7 | 1b | (1b) | | BCP | SR | 1.5E6 | 28 |  | 1145 | D168E (96%) | M28L (85%), Q30R/H (51%/34%) |  | - |
| P8 | 1a | (1a) | | TPV | SR | 3.8E6 | 24 |  | 1599 |  | M28L, Q30R |  | - |
| P9 | 1 | (1a) | | TPV | SR | 2.6E6 | 24 |  | 1663 |  | M28V (10%), Q30H (61%) |  | - |
| P10 | 1a | (1a) | | TPV | SR | 3.0E5 | 48 |  | 1186 |  |  |  | - |
| P11 | 1 | (1a) | | TPV | SR | 2.2E6 | 24 |  | 374 |  |  |  | - |
| P12 | 1a | (1a) | | TPV | SR | 1.3E5 | 24 |  | 337 |  |  |  | - |
| P13 | 1b | (1b) | | TPV | SR | 1.4E6 | 24 |  | 7522 | S122T, I132V (80%) |  |  | - |
| P14 | 1a | (1a) | | TPV | R | 1.7E7 | 24 |  | 4048 |  | M28L, Q30R, L31F |  | **V36M, R155K** |
| P15 | 1 | (1a) | | TPV | R | 3.9E6 | 3 | SE | 10042 | Q80K |  |  |  |
| P16 | 1a | (1a) | | BCP | R | 1.4E5 | 28 | SE | 288 |  |  |  |  |
| P17 | 1b | (1b) | | BCP | R | 1.7E5 | 39 | SE | 217 | S122T, I132V |  |  | S122N |
| P18 | 1a | (1a) | | TPV | R | 3.6E5 | 14 | SE | 35 | S122G | M28L, Q30R |  | **V36M, R155K** |
| P19 | 1 | (1a) | | BCP | R | 2.3E6 | 48 |  | 12272 |  |  |  |  |
| P20 | 1 | (1a) | | TPV | R | 5.4E5 | 24 |  | 1285 |  | Q30R (74%) | M426L |  |
| P21 | 1a | (1a) | | TPV | R | 2.0E6 | 6 | SE | 343 | Q80K |  |  |  |
| P22 | 1a | (1a) | | BCP | NR | 1.0E7 | 13 | NR | 6076 |  | M28L, Q30R |  |  |
| P23 | 1a | (1a) | | BCP | NR | 1.8E6 | 14 | NR | 5028 | **T54S, V55I** |  |  |  |
| P24 | 1a | (1a) | | TPV | NR | 2.3E5 | 4 | NR | 624 |  |  |  | **V36M, R155K** |
| P25 | 1b | (1b) | | TPV | NR | 1.3E6 | 13 | NR + SE | 3961 |  |  |  |  |
| P26 | 1a | (1a) | | BCP | NR | 9.2E6 | 15 | NR | 32633 |  | M28L, Q30R | M426L |  |
| P27 | 1a | (1a) | | TPV | NR | 6.0E5 | 14 | NR + SE | 2598 | V55A |  |  | **V36M, R155K** |
| P28 | 1a | (1a) | | BCP | NR | 2.8E6 | 11 | NR + SE | 3662 | S122G |  |  |  |
| P29 | 1a | (1a) | | TPV | NR | 3.1E6 | 13 | NR | 22654 | Q80K, S122G |  |  | **V36M, R155K** |
| P30 | 1a | (1a) | | BCP | NR | 2.6E6 | 14 | NR | 10289 |  |  |  | **V36M** |
| P31 | 1b | (1b) | | TPV | NR | 1.5E6 | 14 | NR | 439 |  |  |  |  |
| P32 | 1b | (1b) | | BCP | NR | 3.7E6 | 13 | NR | 6538 | Q80M (96%), I132V (90%) | M28L, Q30R, Y93H (18%) | L159F (88%), C316N |  |
| P33 | 1a | (1a) | | TPV | NR | 4.6E5 | 26 | NR + SE | 1532 |  | M28L, Q30R |  | **R155K** |

**Supplementary Table 2: Pre and post treatment resistance detected by ve-SEQ**

Patients received combination therapy including Boceprevir (BCP, n=14) or Telaprevir (TPV, n=19). Thirteen patients achieved sustained virological response (SVR), 8 relapsed (R) and 12 patients were non-responders (NR). Nine patients terminated treatment early because of side effects (SE). Polymorphisms at the following positions were considered potential resistance associated variants (RAVs); **NS3**: V36, Q41, F43, T54, V55, Q80, R109, S122, I132, R155, A156, D168; **NS5a**: M28, Q30, L31, Y93; **NS5b**: S282, L112, L159, C316, L419, R422, M423, M426, I482, V494. Percentages (%) show the frequency of variants; where no percentage is shown the corresponding RAV was detected in all reads. RAVs to the prescribed drug are shown in bold. All NS3 RAVs first detected at any frequency, at any time after treatment failure, are shown in the final column.
