# Supplementary material for: ve-SEQ: Robust, unbiased enrichment for streamlined detection and whole-genome sequencing of HCV and other highly diverse pathogens
Source: F1000Res. 2015 Oct 13;4:1062. [Version 1] doi: 10.12688/f1000research.7111.1 (PMC4821293; doi:10.12688/f1000research.7111.1)
Supplement: Peer review history

# Peer review: Referee response for version 1

**DOI:** 10.5256/f1000research.7657.r12905

**Published:** 2016-04-18

**Authors:** Tanya L Applegate, Rowena Bull

**Affiliations:** 1 Viral Hepatitis Clinical Research Program, The Kirby Institute, University of New South Wales, Sydney, NSW, Australia; 2 Inflammation and Infection Research Centre, University of New South Wales, Sydney, NSW, Australia

Bonsall *et al.* describe an improved metagenomic approach for sequencing HCV, which is adequately described by the title “-ve-SEQ: Robust,unbiased enrichment for streamlined detection and whole-genome sequencing of HCV and other highly divers pathogens”.  The article provides proof-of principle data to the detection of known HCV associated resistance associated variants in a small number of subjects across a range of genotypes. This is a valuable addition to the limited repertoire of sequencing methods available for full HCV genome sequencing.

The article is clearly written, the abstract provides an accurate summary of the article and the overall conclusions are justified on the basis of the results. However, considering this manuscript describes a method, the paper does need more detail regarding the description of methods (including additional information outlining the probe design) and results would allow the reader to reproduce the data, draw their own conclusion and add value to this paper. Additional specific comments are listed below for the authors consideration.

Introduction:

- First paragraph – the authors might wish to consider adding ‘reinfection’ as major challenge to controlling the HCV epidemic. Many of the at-risk populations become re-infected with HCV which will limit long-term DAA success and also necessitate good robust sequencing techniques that distinguish reinfection from relapse.
- 4th paragraph – the authors may wish to consider the recent publication by Bull *et al.* BMC Genomics 2016 in this discussion. It offers a slight improvement to some of the other amplification based methods, including detection of co-infection, but as correctly stated but the authors is still, as are all targeted based amplification methods, biased by primer design.
- Typo in the following statement “with relatively high VLs (>1.8 105 IU/ml) ” need an ‘x’.

Methods:

- Given that this is a methods paper, the methods lack sufficient detail and the probe design is vague. For example, how was the reference sequence for each genotype selected? Is this a prototype strain, in which case genbank ID should be provided or a constructed genotype consensus sequence? I assume that each set in the 4 sets of probes represents a specific genotype.
- Samples selection: Could the authors provide more detail on the criteria by which a "representative" 1a sequence was selected?  The authors show a single comparison of how the 1a probe set compares with the other subtypes / genotypes but it is hard to estimate how this might perform in the real world. The authors mention "informal testing of  a typical sample", but more information is required to the support claims in the paper.
- Limit of detection: The authors should define the level of threshold called as “no resistance”. What was the lowest percentage threshold considered reliable to call variants?  What is the minimum viral load at which this quantification is reliable?

Results:

- Sequence success: Could the authors please clarify that only 29 samples were tested and all 29 were successfully sequenced.
- Cost breakdown: In regards to the statement “n its standard form, metagenomic sequencing of a batch of up to 96 samples costs <£100 per sample.” As the reduced cost re. rationale probe design is discussed as one of the main advantages of this method, could the authors please clarify exactly what is included in the ‘standard metagenomics cost’ and provide a disaggregation of the costs. Ie., is it just the cost of sequencing 96 samples on the HiSeq or did they also include library prep costs in that cost estimate. Is there an estimate of the number of samples that would be required to make this a cost-effective compared in comparison to bulk sequencing the NS3-NS5B regions?
- Figure 2 needs a key to describe the heat map (does Yellow = higher entropy) and more detailed description of how genetically" distinct" genotype 1a is in panel c.
- Probe design: Did the authors in their probe design consider or attempt to target the relatively conserved sequence after the poly U/C tract at the 3’ end of the genome? Perhaps it is too short or lack of reliable sequence for probe design? For while the 3’UTR is unlikely to be of interest for RAV analysis it has been proposed to be important in viral pathogenesis and induction of innate immunity and the exclusion in obtaining 3’UTR sequence in this method does present a small limitation for a subset of viral diversity studies.
- The authors mention "higher read depths" are required for precise characterisation of minor variants, could the authors describe the minimum number of reads that would be required for this?
- The authors refer to a database from which they got their 482 reference whole-genome sequences from which they designed their probes? What is this database and is it publicly available?
- In supplementary table 1 what does probe panel “G123456” mean? According to methods probes were only designed on Gt 1,2,3 and 4….. Ok now that I have continued reading the results I now understand as the design of G123456 probes is described in the results on page 6. I suggest that a section outlining the expanded probe design be added to the methods section so that there is not confusion in understanding the tables. This expanded probe design offers much more potential than the original probe design and needs highlighting.
- Figure 4: - Figure 4 labeling and description in the text needs to be improved to allow the reader to follow which subject is being described.
- Figure 4a: was reinfection with a different variant ruled out? For example what was the genetic distance of the consensus variants between pre-treatment and relapse? What percentage where the RAVs present after treatment?
- The authors indicate NS5A variants were found, but don't provide any more data (what RAVS, what level they were found).
- Sup figures 2 and 3: results look great but it would help for comparison of the different probe panels if the right y-axis was put on the same scale, as has been done for the left y-axis. This is unlikely to be possible with figure 2 as there is an order of magnitude difference between the plots.
- For the RAV analysis which probe set was used?

Discussion:

- First sentence – the reviewers agree that the method is a valuable improvement in comparison to other metagenomic approaches but it still does have some limitations (and some advantages as already discussed in intro) when compared to amplicon approaches and these should be acknowledged and discussed. Specifically, sequencing samples with low viral loads, and the detection of low frequency RAVs is currently more sensitive with targeted amplicon approaches.
- Sensitivity: There is a potential sensitivity issue that has not been addressed with this assay in regards to RAVs as mentioned in the results section. Unless the authors add data to show high sensitivity then this should be discussed in paragraph 2 of the discussion.
- Minor point, Paragraph 3 – “none is as diverse as HCV” – I would change this to “few are as diverse as HCV”. It is debatable depending on your classification but the Enteroviruses are an extremely diverse group…

We have read this submission. We believe that we have an appropriate level of expertise to confirm that it is of an acceptable scientific standard, however we have significant reservations, as outlined above.

# Peer review: Referee response for version 1

**DOI:** 10.5256/f1000research.7657.r12385

**Published:** 2016-04-01

**Authors:** Nicholas J. Loman

**Affiliations:** 1 Institute of Microbiology and Infection, University of Birmingham, Birmingham, UK

This is a potentially important protocol for sequencing viral genomes using an adapted nucleic acid bead capture method. The results are impressive and I think the technique is likely to be useful to many who are undertaking viral sequencing.

The article is written well and I believe acceptable for indexing in its current version.

I have a number of suggestions that may improve the utility of the article to readers:

- I would appreciate a longer description of the NEBNext protocol - I assume this is first strand cDNA synthesis using random hexamers, but this would be useful to spell out.
- The main innovation is the use of IDT xGen Lockdown protocol, but the protocol is not described in much detail. I would appreciate a flowchart or textual description of the protocol, because I would like to know how long it takes, what steps are involved and what equipment is needed to carry it out
  .
- The costs of the per-sample sequencing is given, I would like to see this broken down by component.
- I have a major issue with Figure 2 as presented, due to the use of multiple Y-axes which I think makes it very very hard to interpret. Please split this out into panels with enrichment and unenriched data presented separately. Also please decide on consistent use of scientific notation or regular numbers (I prefer the latter). In fact rather than reporting read depth it would be more informative to report as a fraction of the number of reads from that barcode.
- I'm afraid I can't get on with the "ve-Seq" name, because I read it like "negative-Seq" each time! The authors might consider a more informative and easier to communicate name.
- The authors may consider citing some of the recently published pan-viral capture papers such as VirCAP-Seq ^1^ and relating ve-Seq to this technique.

I have read this submission. I believe that I have an appropriate level of expertise to confirm that it is of an acceptable scientific standard.

## References

[1] BrieseTKapoorAMishraNJainKKumarAJabadoOJLipkinWI: Virome Capture Sequencing Enables Sensitive Viral Diagnosis and Comprehensive Virome Analysis. *MBio*.2015;6(5) : 1062 e01491-15 10.1128/mBio.01491-15 26396248PMC4611031

# Peer review: Referee response for version 1

**DOI:** 10.5256/f1000research.7657.r10798

**Published:** 2015-10-26

**Authors:** Fabio Luciani

**Affiliations:** 1 School of Medical Sciences, University of New South Wales, Sydney, NSW, Australia

In this original research work the authors have developed a novel method for detection and sequencing of HCV genomes from clinical samples adopting a DNA probe approach.

Similar methods have been developed previously for targeted genomes. This approach has been adapted for HCV and has great potential to be applied to other RNA viruses. Overall, in my opinion, this paper is an interesting and novel application which resolve few long standing issues with identification and sequencing of complex viral populations from clinical samples. 

It would be very helpful for the readers if the authors would address the comments below.

1. It would be helpful to have more details on how the first set of probes were chosen. The authors state that these were 155-157 probes, each of length 120t, which roughly equate to 2 full HCV genomes. Can the authors describe what exactly was the algorithm to identify those fragments from the total genomes considered?
  It is clear that the second set for the rare GT were constructed with 80% dissimilarities from the first set.
2. The critical message is that this approach seems to break the barrier of sequencing very low viral loads in an unbiased approach. I found this a very important result. It is however clear from the data that the attempt is not fully successful as only partial genomes are obtained. Maybe some more clear statements highlighting where we are up to with this method and what can be done to improve. I would recommend to have Supp Figure 1 in the main text as this is a rather interesting result showing that there is a better enrichment for low viral loads.
3. I don't fully agree with the authors with the conclusions.
  This method is still not reliable in terms of detecting near-full length genome at low viral load, and therefore the classical PCR-primer genotype specific primers are needed.
  Rather, I would encourage the authors to discuss more the implication of such an approach (and improved ones into the future) for sequencing more complex scenarios, such as recombination, multiple infections, reinfections, superinfection etc.
4. A comment that I hope will generate some feedbacks
  DAA treatment are much better than those considered in this manuscript. HARVONY and GS-5816 are breaking the barrier of 95% SVR pan genotype. 
  This is the first time in history of antiviral therapy of such a limited drug resistance.
  I think the proposed method will have higher chances to be applied in other settings (as mentioned before to study complex genomic rearrangements)
  Maybe worth thinking about this.
5. Finally, this work made me also wonder on what limitations still exist that this method does not assess. It would be interesting to mention that for understanding viral evolution, including drug resistance, there is need to identify compensatory mutations and epistatic interactions, which may occur between viral mutations that are far apart in the genome.
  This is a problem of haplotype reconstruction which has been proven to be very difficult to solve if the staring points are short reads. 
  Thank you for the opportunity to comment on such a novel and interesting work.

I have read this submission. I believe that I have an appropriate level of expertise to confirm that it is of an acceptable scientific standard.
